# Prevalence, incidence, and complications of malnutrition in severely injured patients

**DOI:** 10.1007/s00068-024-02711-8

**Published:** 2025-01-24

**Authors:** Esmee A. H. Verheul, Suzan Dijkink, Pieta Krijnen, Jochem M. Hoogendoorn, Sesmu Arbous, Ron Peters, George C. Velmahos, Ali Salim, Daniel D. Yeh, Inger B. Schipper

**Affiliations:** 1 Department of Trauma Surgery, Leiden University Medical Center, Post zone K6-R, P.O. Box 9600, Leiden, 2300 RC The Netherlands; 2 Department of General Surgery, Haaglanden Medical Center, The Hague, The Netherlands; 3 Acute Care Network West Netherlands, Leiden, The Netherlands; 4 Department of Intensive Care, Leiden University Medical Center, Leiden, The Netherlands; 5 Department of Intensive Care, Haaglanden Medical Center, The Hague, The Netherlands; 6 Department of Trauma Surgery, Massachusetts General Hospital, Boston, MA USA; 7 Department of Surgery, Brigham and Women’s Hospital, Boston, MA USA; 8 Department of Surgery, Denver Health Medical Center, Denver, CO USA

**Keywords:** Malnutrition, Severe injury, Complications, Severely injured, Intensive care unit

## Abstract

**Background:**

Severely injured patients may suffer from acute disease-related or injury-related malnutrition involving a marked inflammatory response. This study investigated the prevalence and incidence of malnutrition and its relation with complications in severely injured patients admitted to the intensive care unit (ICU).

**Methods:**

This observational prospective cohort study included severely injured patients (Injury Severity Score ≥ 16), admitted to the ICU of five level-1 trauma centers in the Netherlands and United States. Malnutrition was defined as a Subjective Global Assessment score ≤ 5. Complications included systemic-, surgery-, and fracture-related complications, pneumonia, urinary tract infection, deep venous thrombosis, and pulmonary embolism. In-ICU and in-hospital mortality were recorded separately. The complication rate was compared between patients who had or developed malnutrition and patients who remained well-nourished, using multivariable logistic regression analysis.

**Results:**

Of 100 included patients, twelve (12%) were malnourished at admission. Of the 88 well-nourished patients, 44 developed malnutrition during ICU admission, (ICU incidence 50%, 95% confidence interval [CI] 40–60%). Another 18 patients developed malnutrition at the ward (overall in-hospital incidence 70%, 95% CI 61–80%). The 62 patients who developed malnutrition and 12 patients who were malnourished upon admission had more complications than the 26 patients who remained well-nourished (58% vs. 50% vs. 27% respectively; *p* = 0.03; Odds Ratio 3.4, 95% CI 1.2–9.6).

**Conclusions:**

50% of severely injured patients developed malnutrition during ICU admission, increasing to 70% during hospital admission. Malnutrition was related to an increased risk of complications. Recognition of sub-optimally nourished severely injured patients and assessment of nutritional needs could be valuable in optimizing their clinical outcomes.

**Level of evidence:**

Level III, Prognostic/Epidemiological.

## Key points

This observational prospective cohort study investigated the prevalence and incidence of malnutrition and its relation with complications and in-hospital outcomes in severely injured patients admitted to the ICU. The majority of severely injured patients developed malnutrition during ICU and hospital admission. Malnutrition is found to be correlated to an increased risk of complications, especially pneumonia, and a longer hospital stay.

## Background

Malnutrition is a common but frequently unrecognized problem in hospitalized patients, despite its association with adverse outcomes, such as infections, prolonged hospital stay, impaired wound healing, and mortality [1–4]. According to the American Society for Parenteral and Enteral Nutrition (A.S.P.E.N.) three types of malnutrition are defined based on etiology, including social and environmental circumstances, chronic illness, and acute illness [4]. Severely injured patients may suffer from acute disease-related or injury-related malnutrition involving a marked inflammatory response [4]. Because of the stress response following traumatic injuries, severely injured patients often endure an altered metabolic state in order to preserve energy for vital tissues. This can cause a deterioration of the nutritional status, which again negatively influences the stress and metabolic response after trauma [5]. Due to this vicious circle of deterioration of the nutritional- and health status, severely injured patients are at risk for considerable additional harm from malnutrition. Current estimates of the in-hospital prevalence of malnutrition at admission in severely injured patients range from 7 to 76%, depending upon the setting, population, and nutritional assessment tool used [5].

Recognition of sub-optimally nourished severely injured patients and assessment of their nutritional needs is crucial in order to improve their clinical outcomes. Despite the increasing number of studies on malnutrition in hospitalized patients, little is known about the risk of developing malnutrition during hospital admission and its consequences in the severely injured patient population. The goal of this study was to determine the prevalence and incidence of malnutrition, and the relation with complications in severely injured patients who are admitted to the intensive care unit (ICU).

## Methods

The Malnutrition in Polytrauma Patients (MaPP) study is an observational prospective cohort study that was performed at five Level-1 trauma centers, three in the United States and two in the Netherlands. All consecutive adult (≥ 18 years) patients with severe injuries (Injury Severity Score, ISS ≥ 16), caused by blunt trauma, who were admitted to the ICU of one of the participating centers were eligible for inclusion. Patients must be admitted to the ICU for more than 48 h and should not be primarily managed in another hospital. Patients with burn wounds and penetrating injuries were excluded because the factors influencing prognosis and treatment differ significantly from those in blunt trauma patients, and it was anticipated that there would not be sufficient cases to conduct subanalyses. Written informed consent was obtained from the patients or their legal representative on the day of ICU admission or as soon as possible after that day. The study was conducted according to the guidelines of the Declaration of Helsinki and approved by the local Institutional Review Boards (protocol number: NL64016.058.17). The study is described in detail in the published study protocol [6]. Patient inclusion in the Netherlands began in July 2018 and concluded in April 2022, while in the United States, it started in May 2018 and ended in February 2020. This study has been reported in line with the Strengthening the Reporting of Observational Studies in Epidemiology (STROBE) Statement [7].

### Sample size

As described in the study protocol, the a priori sample size calculation showed that 195 patients were needed to show a difference in complication rate between the groups with and without malnutrition [6]. However, due to the low inclusion rate during the COVID-19 pandemic, it was decided to prematurely end the inclusion at 100 patients.

### Study parameters

#### Nutritional status

**Fig. 1:**
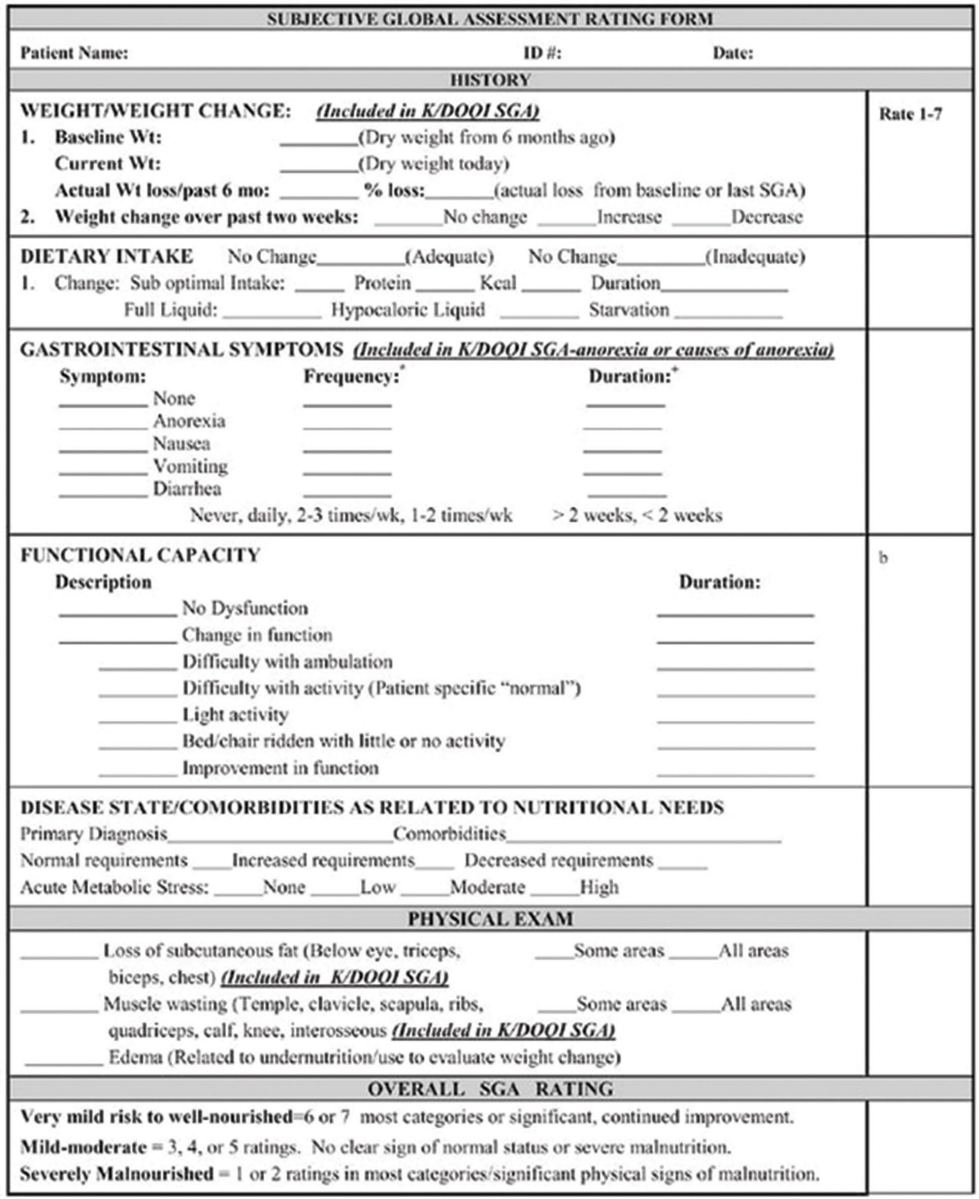
Subjective Global Assessment rating form [8]

The Subjective Global Assessment scale (Fig. 1) was used to assess the nutritional status and determine pre-existent and in-hospital developed malnutrition [8]. The SGA scale is a nutritional assessment tool that has been validated for the acute hospital setting, for surgical patients and for patients admitted to the ICU requiring mechanical ventilation [8–10]. The SGA evaluates weight change (over the past 2 weeks and 6 months), in-/ adequate dietary intake change, gastrointestinal symptoms (less appetite, nausea, vomiting, diarrhea), and functional capacity (dysfunction, bedridden, difficulty with normal activities). Determining the SGA score also includes a physical examination of subcutaneous fat loss (eyes, triceps, biceps) and muscle wasting (e.g., clavicle, knee, shoulder, and quadriceps). The SGA is scored on a scale ranging from 1 to 7. Patients are classified as A (well-nourished; scores 6–7), B (mild to moderately malnourished; scores 3–5) or C (severely malnourished; scores 1–2) [11]. In this study, B and C were combined in one category (malnourished, defined by an SGA score ≤ 5) [6]. The SGA was scored by trained personnel at ICU admission, every five days during ICU admission, at ICU discharge, every week on the ward, and at hospital discharge. A recent systematic review indicated that the SGA score can be used to assess in-hospital acquired malnutrition [12].

#### Other parameters and in-hospital outcomes

Information on nutritional support was collected, and patients were categorized based on whether they received oral feeding or (par)enteral feeding. In the patients who received (par)enteral nutrition, it was documented whether nutrition was initiated within 48 h or after 48 h of admission. Target energy goals were calculated through a weight-based predictive Eq. (25 kcal/kg/day). In overweight patients (BMI > 25 kg/m^2^), the ideal body weight was used, which is calculated by the following equation: 0.9 x height in cm − 100 (male) (or − 106 (female)) [13]. According to the ESPEN guidelines, target energy goals should be met after 3–7 days of admission. It was documented whether goals were met after < 48 h, 3–7 days, and after > 7 days of admission. Albumin and pre-albumin levels were measured within 24 h of admission. Surgical procedures that required patients to go to the operating room were documented. The following complications were included in the analysis: systemic complications (sepsis, Acute Respiratory Distress Syndrome (ARDS), Systemic Inflammatory Response Syndrome (SIRS), multiple-organ failure), surgery-related complications (anastomotic leak, stoma surgical site infection deep and superficial, abscess, (re)bleeding, wound infection), pneumonia, urinary tract infection (UTI), deep venous thrombosis (DVT), pulmonary embolism (PE), and fracture-related complications (compartment syndrome, thromboembolic disease, fat embolism syndrome, reoperation due to non-union or mal-union). Pneumonia was defined as lung inflammation caused by a bacterial or viral infection. Consequently, COVID-19 pneumonia was also classified as pneumonia. Furthermore, in-ICU and in-hospital mortality were included in the analysis. Other in-hospital outcomes included hospital length of stay (LOS), ICU LOS, and ventilator days.

### Statistical analysis

**Fig. 2:**
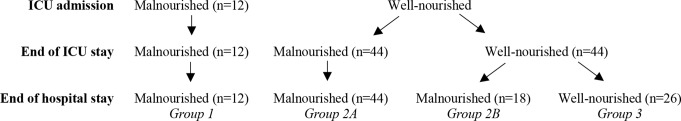
Distribution of severely injured patients according to their nutritional status based on the Subjective Global Assessment (SGA)

Statistical analyses were performed with IBM SPSS Statistics for Windows, version 25. P-values < 0.05 were considered statistically significant. The baseline characteristics and outcomes of the patients who remained well-nourished throughout hospital admission (Group 1; Fig. 2), patients who became malnourished during hospital admission (Group 2), and patients who had malnutrition at hospital admission (Group 3) were compared using the Chi-Square test for categorical variables and the one way ANOVA test for continuous variables. Furthermore, the baseline characteristics and outcomes of the patients who became malnourished during ICU admission (Group 2 A; Fig. 2) were compared to those of the patients who became malnourished during admission to the ward (Group 2B) using the Fisher’s exact test for categorical variables and the independent samples T-test for continuous variables.

The *prevalence of pre-existing malnutrition* was calculated as the proportion (with 95% confidence interval [CI]) of patients who were malnourished at ICU admission. The *incidence of in-ICU malnutrition* was calculated as the proportion (with 95% CI) of patients who became malnourished during ICU stay. The *incidence of in-hospital malnutrition* was calculated as the proportion (with 95% CI) of patients that became malnourished during total hospital stay.

The complication rate was calculated as the proportion of patients with any of the included complications during hospital admission. The complication rate was compared between patient groups using the Chi-square test. The odds ratio (OR) (with 95% CI) of complications during hospital stay for patients with malnutrition (Groups 1 and 2; Fig. 2) compared to well-nourished patients (Group 3) was calculated. To correct for potential confounders a multivariate logistic regression analysis was performed including the baseline characteristics that differed between the well-nourished and malnourished groups with univariate *p* < 0.10.

## Results

### Prevalence and incidence of malnutrition

The mean age of the 100 included patients was 50 (± 21) years, 70 patients were male (Table 1). Seven patients died during their stay at the Intensive care unit (ICU), and four more patients died while being admitted to the ward. Twelve patients were considered malnourished at ICU admission (SGA score ≤ 5; Group 1; Fig. 2), the prevalence of pre-existing malnutrition being 12% (95% CI 5.6–18.4%). These patients scored insufficient (i.e. ≤ 5 points in SGA item) on weight (loss) (*n* = 8), dietary intake (*n* = 12), gastrointestinal symptoms (*n* = 3), functional capacity (*n* = 1), disease state (*n* = 12), and/or physical exam (*n* = 5). All 12 malnourished patients remained malnourished throughout hospital admission. Of the 88 patients that were well-nourished at admission, 44 became malnourished during ICU stay (Group 2 A; Fig. 2) (incidence of in-ICU malnutrition 50.0%, 95% CI 39.6–60.4%). These 44 patients scored insufficient on weight (loss) (*n* = 32), dietary intake (*n* = 42), gastrointestinal symptoms (*n* = 2), functional capacity (*n* = 14), disease state (*n* = 44), and/or physical exam (*n* = 26). Additionally, 18 patients became malnourished during admission to the ward (Group 2B; Fig. 2). These 18 patients scored insufficient on weight (loss) (*n* = 16), dietary intake (*n* = 14), gastrointestinal symptoms (*n* = 5), functional capacity (*n* = 18), disease state (*n* = 18), and/or physical exam (*n* = 15). In total, 62 patients became malnourished during hospital stay (Group 2; Fig. 2), with an incidence of in-hospital malnutrition of 70.5% (95% CI 60.9–80.0%).

**Table 1:** Patient characteristics according to their nutritional status

	Total (*n* = 100)	Malnourished at admission (*n* = 12; Group 1)	Become malnourished during hospital stay (*n* = 62; Group 2)	Well-nourished throughout hospital stay (*n* = 26; Group 3)	*P* value
Age in years, mean ± SD	50 ± 21	61 ± 25	47 ± 20	52 ± 21	0.09
Male sex, n (%)	70 (70)	10 (83)	40 (65)	20 (77)	0.29
BMI in kg/m^2^, mean ± SD	26 ± 5	26 ± 4	26 ± 5	27 ± 6	0.57
Obesity (BMI ≥ 30 kg/m^2^), n (%)	19 (19)	3 (25)	11 (18)	5 (19)	0.84
Severe injury (AIS ≥ 4), n (%)					
*Head*	44 (44)	3 (25)	32 (52)	9 (35)	0.13
*Chest*	29 (29)	2 (17)	19 (31)	8 (31)	0.60
*Abdomen*	9 (9)	1 (8)	6 (10)	2 (8)	0.95
*Extremity*	14 (14)	1 (8)	10 (16)	3 (12)	0.71
ISS ≥ 25, n (%)	67 (67)	5 (42)	48 (77)	14 (54)	**0.01**
GCS score ≤ 8, n (%)	42 (42)	3 (25)	29 (47)	10 (38)	0.34
Alcohol abuse, n (%)	15 (15)	2 (17)	10 (16)	3 (12)	0.85
Malignancy, n (%)	8 (8)	2 (17)	3 (5)	3 (12)	0.29
Nutrition, n (%)					0.19
*Oral*	29 (29)	5 (42)	14 (23)	10 (39)	
*(Par)enteral*	71 (71)	7 (58)	48 (77)	16 (62)	
Initiation of (par)enteral nutrition, n (%)					0.94
*< 48 h*	63 (89)	6 (86)	43 (90)	14 (88)	
*≥ 48 h*	8 (11)	1 (14)	5 (10)	2 (13)	
Time until target energy goals were met, n (%)					0.33
*< 48 h*	19 (19)	0 (0)	14 (23)	5 (19)	
*3–7 days*	67 (67)	10 (83)	38 (61)	19 (73)	
*> 7 days*	14 (14)	2 (17)	10 (16)	2 (8)	
Albumin level at admission in g/L, mean ± SD	34 ± 7 (*n* = 91)	35 ± 7 (*n* = 11)	33 ± 7 (*n* = 57)	34 ± 8 (*n* = 23)	0.69
Pre-albumin level at admission in g/L, mean ± SD	0.17 ± 0.06 (*n* = 64)	0.15 ± 0.05 (*n* = 6)	0.18 ± 0.06 (*n* = 41)	0.17 ± 0.06 (*n* = 17)	0.29
Surgery, n (%)	82 (82)	7 (58)	55 (89)	20 (77)	**0.03**

AIS, Abbreviated Injury Scale severity (last digit of the AIS code); BMI, Body Mass Index; GCS, Glasgow Coma Scale; ISS, Injury Severity Score; PEG, Percutaneous Endoscopic Gastrostomy; SD, Standard deviation

### Patient characteristics

Patients who became malnourished during their hospital stay (Group 2; Fig. 2) were significantly more likely to have very severe injuries (ISS ≥ 25), than the patients who were malnourished at admission (Group 1) or the patients who remained well-nourished throughout hospital stay (Group 3) (77% vs. 42% vs. 54% respectively; *p* < 0.01; Table 1). Furthermore, a higher percentage of these patients underwent surgery (89%) compared to the patients who were already malnourished (58%) or those who remained well-nourished (77%; *p* = 0.03; Table 1). Comparison between the 44 patients who became malnourished during ICU admission (Group 2 A; Fig. 2) and the 18 patients who became malnourished during admission to the ward (Group 2B) revealed no statistically significant differences (Table 2).

**Table 2:** Patient characteristics of the 62 patients who developed malnutrition during hospital admission

	Total (*n* = 62)	Become malnourished during ICU stay (*n* = 44; Group 2 A)	Become malnourished during admission to the ward (*n* = 18; Group 2B)	*P* value
Age in years, mean ± SD	47 ± 20	47 ± 20	46 ± 19	0.85
Male sex, n (%)	40 (65)	26 (59)	14 (78)	0.24
BMI in kg/m^2^, mean ± SD	26 ± 5	25 ± 4	27 ± 5	0.32
Obesity (BMI ≥ 30 kg/m^2^), n (%)	11 (18)	7 (16)	4 (22)	0.72
Severe injury (AIS ≥ 4), n (%)				
*Head*	32 (52)	25 (57)	7 (39)	0.27
*Chest*	19 (31)	11 (25)	8 (44)	0.14
*Abdomen*	6 (10)	3 (7)	3 (17)	0.34
*Extremity*	10 (16)	7 (16)	3 (17)	1.00
ISS ≥ 25, n (%)	48 (77)	32 (73)	16 (89)	0.20
GCS score ≤ 8, n (%)	29 (47)	24 (55)	5 (28)	0.09
Alcohol abuse, n (%)	10 (16)	8 (18)	2 (11)	0.71
Malignancy, n (%)	3 (5)	2 (5)	1 (6)	1.00
Nutrition, n (%)				0.32
*Oral*	14 (23)	8 (18)	6 (33)	
*(Par)enteral*	48 (77)	36 (82)	12 (67)	
Initiation of (par)enteral nutrition, n (%)				0.31
*< 48 h*	43 (90)	31 (86)	12 (100)	
*≥ 48 h*	5 (10)	5 (14)	0 (0)	
Time until target energy goals were met, n (%)				0.71
*< 48 h*	14 (23)	9 (21)	5 (28)	
*3–7 days*	38 (61)	27 (61)	11 (61)	
*> 7 days*	10 (16)	8 (18)	2 (11)	
Albumin level at ICU discharge in g/L, mean ± SD	33 ± 7 (*n* = 57)	32 ± 7 (*n* = 41)	36 ± 5 (*n* = 16)	0.05
Pre-albumin level at ICU discharge in g/L, mean ± SD	0.18 ± 0.06 (*n* = 41)	0.18 ± 0.06 (*n* = 27)	0.19 ± 0.05 (*n* = 14)	0.38
Surgery, n (%)	55 (89)	39 (89)	16 (89)	1.00

AIS, Abbreviated Injury Scale severity (last digit of the AIS code); BMI, Body Mass Index; GCS, Glasgow Coma Scale; ISS, Injury Severity Score; PEG, Percutaneous Endoscopic Gastrostomy; SD, Standard deviation

### Complications and other in-hospital outcomes

The complication rate during hospital admission was significantly higher in the 62 patients who developed malnutrition (Group 2; Fig. 2) and the 12 patients who were malnourished upon admission (Group 1) compared to the 26 patients who remained well-nourished throughout their hospital stay (Group 3) (58% vs. 50% vs. 27% resp.; *p* = 0.03; Table 3). ICU LOS, number of ventilator days, and hospital LOS were not statistically different between the three groups. No significant difference in ICU-mortality and in-hospital mortality was seen between the patients who became malnourished, those who were already malnourished, and those who remained well-nourished.

**Table 3:** Patient outcomes according to their nutritional status

	Total (*n* = 100)	Malnourished at admission (*n* = 12; Group 1)	Become malnourished during hospital stay (*n* = 62; Group 2)	Well-nourished throughout hospital stay (*n* = 26; Group 3)	*P* value
Complication, n (%)	49 (49)	6 (50)	36 (58)	7 (27)	**0.03**
ICU-mortality, n (%)	7 (7)	0 (0)	3 (5)	4 (15)	0.13
In-hospital mortality, n (%)	11 (11)	1 (8)	5 (8)	5 (19)	0.30
Systemic complications, n (%)	10 (10)	2 (17)	7 (11)	1 (4)	0.41
Surgical complications, n (%)	9 (9)	1 (8)	6 (10)	2 (8)	0.95
Fracture-related complications, n (%)	2 (2)	1 (8)	1 (2)	0 (0)	0.22
Pneumonia, n (%)	40 (40)	4 (33)	30 (48)	6 (23)	0.08
Urinary tract infection, n (%)	11 (11)	1 (8)	8 (13)	2 (8)	0.74
Venous thromboembolism, n (%)	7 (7)	0 (0)	7 (11)	0 (0)	0.10
ICU LOS in days *, mean ± SD	13 ± 18	11 ± 8	14 ± 18	11 ± 23	0.73
Ventilator days *, mean ± SD	8 ± 14	7 ± 8	9 ± 10	9 ± 24	0.91
Hospital LOS in days **, mean ± SD	29 ± 24	25 ± 17	33 ± 26	19 ± 22	0.05

ICU, Intensive care unit; LOS, Length of stay; n, number; SD, standard deviation;

* Patients who died during ICU admission were excluded (*n* = 7)

** Patients who died during hospital admission (*n* = 11) or were transferred to another hospital (*n* = 2) were excluded

Concerning the 62 patients who developed malnutrition during hospital admission, the 44 patients who became malnourished during ICU stay (Group 2 A; Fig. 2) suffered significantly more from pneumonia than the 18 patients who developed malnutrition during admission to the ward (Group 2B; Fig. 2) (59% vs. 22%; *p* = 0.01; Table 4). Furthermore, ICU LOS and ventilator days were significantly higher in the patients who became malnourished during ICU stay than the patients who developed malnutrition during admission to the ward.

**Table 4:** Patient outcomes of the 62 patients who developed malnutrition during hospital admission

	Total (*n* = 62)	Become malnourished during ICU stay (*n* = 44; Group 2 A)	Become malnourished during admission to the ward (*n* = 18; Group 2B)	*P* value
Complication, n (%)	36 (58)	28 (64)	8 (44)	0.26
ICU-mortality, n (%)	3 (5)	3 (7)	0 (0)	0.55
In-hospital mortality, n (%)	5 (8)	5 (11)	0 (0)	0.31
Systemic complications, n (%)	7 (11)	5 (11)	2 (11)	1.00
Surgery-related complications, n (%)	6 (10)	5 (11)	1 (6)	0.66
Fracture-related complications, n (%)	1 (2)	1 (2)	0 (0)	1.00
Pneumonia, n (%)	30 (48)	26 (59)	4 (22)	**0.01**
Urinary tract infection, n (%)	8 (13)	6 (14)	2 (11)	1.00
Venous thromboembolism, n (%)	7 (11)	5 (11)	2 (11)	1.00
ICU LOS in days *, mean ± SD	14 ± 18	17 ± 20	8 ± 5	**0.01**
Ventilator days *, mean ± SD	9 ± 10	10 ± 11	5 ± 5	**0.01**
Hospital LOS in days **, mean ± SD	33 ± 26	35 ± 29	31 ± 18	0.67

ICU, Intensive care unit; LOS, Length of stay; n, number; SD, standard deviation;

* Patients who died during ICU admission were excluded (*n* = 3)

** Patients who died during hospital admission (*n* = 5) or were transferred to another hospital (*n* = 2) were excluded

The crude odds ratio (OR) for complications in malnourished compared to well-nourished patients was 3.3 (95% CI 1.3–8.4). After correction for age, injury severity, and surgery, the increased risk of complications in malnourished patients remained statistically significant (OR 3.4, 95% CI 1.2–9.6).

## Discussion

To our knowledge, this is the first study that analyzed the relationship between in-hospital developed malnutrition and complications in severely injured patients. 12% of all severely injured patients admitted to the ICU were already malnourished at admission. The incidence of in-ICU malnutrition was 50.0% and the incidence of in-hospital malnutrition was 70.5%. Complications occurred significantly more often in malnourished patients than in well-nourished patients.

Several studies have been published concerning the prevalence of malnutrition in trauma patients, as assessed by the SGA at hospital admission. In two studies including trauma patients admitted to the ICU, the prevalence of malnutrition at admission was 11 and 12% [14, 15]. In patients with a moderate-to-severe traumatic brain injury (TBI) admitted to the ICU, 14% were found to be malnourished [16]. These results are comparable to our results. When looking at all trauma patients, both ICU and non-ICU patients, this prevalence ranged up to 48% at admission [17–19]. In studies including geriatric trauma patients, 30–66% were malnourished at hospital admission [20–22]. Since malnutrition is more common in the elderly population, this probably explains the higher prevalence of malnutrition in these study groups [23].

Studies reporting the changes in the nutritional status of trauma patients also found a significant increase in malnutrition during hospital admission. In acute care surgery patients, 27% were malnourished at admission and 41% was malnourished after one week of admission [24]. In a study by Chapple et al. concerning ICU patients with moderate TBI (GCS 9–12) or severe TBI (GCS 3–8), malnutrition increased from 14% at admission to 44% at hospital discharge [16]. We found that the incidence of in-hospital malnutrition was 70.5%, with a prevalence of malnutrition of 74% at hospital discharge. A higher Injury Severity Score (ISS) is found to be related to higher levels of proinflammatory cytokines, such as tumor necrosis factor-α (TNF-α) and interleukin-6 (IL-6) [25]. These proinflammatory cytokines can cause the body to be in a hypermetabolic state and therefore lead to a loss of body resources [5]. In addition, severely injured patients might suffer more from gastro intestinal-problems such as an ileus, or have to undergo surgery more frequently than TBI patients, which could cause a deterioration in the nutritional status. This might explain the higher incidence of in-hospital malnutrition in our severely injured patient study group compared to the isolated TBI population of Chapple et al. [16].

The high incidence of malnutrition is not simply a matter of insufficient emphasis on nutritional support in the five included hospitals, as the ICU protocols of the five included hospitals align with the ESPEN recommendations [13]. According to these guidelines, (par)enteral nutrition ((P)EN) should be initiated within 48 h if oral intake is not possible. In our patient group, 89% of the (P)EN was initiated within 48 h. Reasons for not starting P(EN) within 48 h were: septic shock (*n* = 1), gastric retention (*n* = 2), or fasting before multiple surgeries (*n* = 5). In these 8 patients, (P)EN was initiated between 48 and 96 h after admission. Furthermore, ESPEN recommends that full (P)EN (i.e. meeting 100% of caloric needs) shall be prescribed within three to seven days to prevent overfeeding. However, 19 patients (19%) received full (P)EN within 48 h and 14 patients (14%) did not meet caloric needs within 7 days. This was not statistically significant between the patients who were malnourished or developed malnutrition and the patients who remained well-nourished. Possibly, the hypermetabolic catabolic state following severe trauma cannot be sufficiently compensated so that a deterioration in nutritional status can be prevented in all cases, even with adequate nutritional therapy. Additionally, the unavoidable fasting period before surgery and the resulting acute phase response after surgery make polytrauma patients exceptionally susceptible to malnutrition. Studies on developments related to peri-operative management are regularly published, such as the Enhanced Recovery After Surgery (ERAS) protocol [26]. One component of the ERAS protocol is early oral feeding after surgery (starting 4 h post-surgery). This approach can lead to faster intestinal recovery, shorter postoperative hospital stays, and fewer complications for patients undergoing gastrointestinal surgery [27]. Since polytrauma patients frequently have multiple surgeries within the initial days of ICU admission, careful monitoring of enteral nutrition and close collaboration with a dietitian is essential for managing both the timing and quantity of enteral feeding.

However, providing more nutrition is not always beneficial, as overfeeding is known to pose risks for ICU patients [28]. Overfeeding can lead to complications such as hyperglycemia, increased carbon dioxide production (leading to respiratory complications), and fat accumulation in the liver, especially in critically ill patients [29]. The endogenous glucose production is elevated in the early phases of critical illness due to stress-induced metabolic changes, which makes patients particularly vulnerable to overfeeding during this time [30]. Indirect calorimetry is a tool that measures oxygen consumption (VO2) and carbon dioxide production (VCO2) to calculate a patient’s actual energy expenditure [31]. This allows healthcare providers to tailor nutritional interventions more precisely, avoiding the potential risks of both underfeeding and overfeeding, especially during ICU admission. On the other hand, following ICU admission, calorie and protein requirements typically rise as patients become more physically active and mobilized during their transition to the ward [32]. However, nutritional intake during this phase may fall short of meeting the increased demands, leaving severely injured patients vulnerable to malnutrition, even while admitted to the ward. Personalized nutrition plans, based on each patient’s metabolic needs, can improve recovery outcomes and reduce complications associated with improper feeding strategies.

In earlier publications, malnutrition, defined as SGA ≤ 5, was found to be related to increased mortality, complications, and prolonged hospital LOS in trauma patients [17, 18, 24]. In critically ill patients, a significant association was demonstrated between SGA and mortality, pressure injuries, length of stay, and ICU readmission rates [10, 33, 34]. Our study found a relationship between malnutrition and complications (Table 3). Surprisingly, ICU mortality and in-hospital mortality seemed higher in the well-nourished patients compared to the patients who were malnourished or developed malnutrition (15% vs. 0% vs. 5%, *p* = 0.13; 19% vs. 8% vs. 8%, *p* = 0.30, resp.), although both differences were not statistically significant (Table 3). This apparent contradictory result may be due to the fact that deterioration in nutritional status occurs gradually and can take several days. Of the 11 patients who died during hospital admission, 6 died within the first week of admission. It seems unlikely that their nutritional status could have deteriorated that much during the short period until their passing, resulting in a higher percentage of patients who were still well-nourished at the time of death. Concerning other in-hospital outcomes among patients who survived their admission, such as ICU LOS, ventilator days, and hospital LOS, no difference was found in the patients that were malnourished or developed malnutrition during admission and the patients who were well-nourished (Table 3). Although the hospital LOS seemed longer for the patients who became malnourished (Table 3), this difference was not statistically significant (*p* = 0.05), possibly due to a lack of statistical power. Lastly, patients who developed malnutrition during their ICU stay experienced significantly higher rates of pneumonia, had a longer ICU length of stay, and required more ventilator days compared to those who became malnourished during ward admission (Table 4). Thus, malnutrition seems to be evidently correlated with complications and in-hospital outcomes. However, the causal relationship between malnutrition and these outcomes remains ambiguous, as both have the potential to influence the other. For example, malnutrition can make a person more susceptible to infection, and infection also contributes to a deterioration of the nutritional status [35]. In addition, malnutrition at admission is known to be associated with prolonged hospital LOS [36]. Furthermore, the longer a patient stays in a hospital, the higher the probability of acquiring an infection [37]. In conclusion, malnutrition seems to be evidently correlated with complications and in-hospital outcomes, but the causal correlation cannot be established.

### Limitations

Since not much nutritional research has been done on severely injured patients, this study can be considered one of the largest studies on the subject. The sample size was limited to 100 patients for pragmatic reasons. Not all patients who were considered eligible for the study were included. The primary reasons for this were organizational challenges as the study demanded significant time from ICU staff, and difficulties in obtaining informed consent (which can be considered burdensome for families of critically ill patients). However, we do not believe that this has led to selection bias in the included patient group. Although the difference in the overall complication rate between the patient groups was statistically significant, the statistical power was too low to detect clinically relevant differences for specific complications, for instance for in-hospital mortality, pneumonia, and venous thromboembolism. Another limitation is presented by the fact that there is no ‘gold standard’ for assessing nutritional status. We used the SGA, as it has been validated for ICU patients and is proven to be the most predictive for outcomes. The SGA score itself, however, is not very discriminative, since the difference between an SGA score of 5 (malnourished) or 6 (well-nourished) can be very minimal. To increase reliability and reduce interobserver variability, the SGA scores were verified by one investigator at the end of data collection. Unfortunately, not enough patients with severe malnutrition (SGA ≤ 2) were included to perform a separate analysis for SGA groups. Therefore, no distinction was made in the severity of malnutrition; SGA scores of 1 to 5 all reflected a malnourished status. Lastly, as already stated in the discussion section, the causal correlation between malnutrition and both complications and in-hospital outcomes cannot be established, since these components are interdependent.

## Conclusion

Over 50% of all well-nourished severely injured patients develop malnutrition during ICU admission, increasing to 70% during their total hospital stay. Malnutrition in severely injured patients developed during ICU and hospital admission is found to be related to an increased risk of complications. Therefore, awareness of the importance of nutritional strategies needs to become a common ground for all clinicians treating severely injured patients. Recognition of sub-optimally nourished severely injured patients and assessment of their nutritional needs is crucial in order to improve their clinical outcome.

## Data Availability

Data is provided within the manuscript.
